# Development of early mathematical skills with a tablet intervention: a randomized control trial in Malawi

**DOI:** 10.3389/fpsyg.2015.00485

**Published:** 2015-04-23

**Authors:** Nicola J. Pitchford

**Affiliations:** School of Psychology, University of NottinghamNottingham, UK

**Keywords:** tablets, technology, primary school, mathematics, intervention, evaluation, randomized control trial

## Abstract

Evaluation of educational interventions is necessary prior to wide-scale rollout. Yet very few rigorous studies have been conducted on the effectiveness of tablet-based interventions, especially in the early years and in developing countries. This study reports a randomized control trial to evaluate the effectiveness of a tablet intervention for supporting the development of early mathematical skills in primary school children in Malawi. A total sample of 318 children, spanning Standards 1–3, attending a medium-sized urban primary school, were randomized to one of three groups: maths tablet intervention, non-maths tablet control, and standard face-to-face practice. Children were pre-tested using tablets at the start of the school year on two tests of mathematical knowledge and a range of basic skills related to scholastic progression. Class teachers then delivered the intervention over an 8-weeks period, for the equivalent of 30-min per day. Technical support was provided from the local Voluntary Service Overseas (VSO). Children were then post-tested on the same assessments as given at pre-test. A final sample of 283 children, from Standards 1–3, present at both pre- and post-test, was analyzed to investigate the effectiveness of the maths tablet intervention. Significant effects of the maths tablet intervention over and above standard face-to-face practice or using tablets without the maths software were found in Standards 2 and 3. In Standard 3 the greater learning gains shown by the maths tablet intervention group compared to both of the control groups on the tablet-based assessments transferred to paper and pencil format, illustrating generalization of knowledge gained. Thus, tablet technology can effectively support early years mathematical skills in developing countries if the software is carefully designed to engage the child in the learning process and the content is grounded in a solid well-constructed curriculum appropriate for the child’s developmental stage.

## Introduction

Research with digital educational software has shown increased motivation (Rosas et al., 2003) and promotion of positive attitudes (Ke, 2008) toward mathematics in primary school children. However, a recent study has concluded that although technology is used in many classrooms in the West, its potential to support learning is often underutilized due to limitations in its design and content (Yelland and Kilderry, 2010). Consequently, findings regarding the attainment benefits of technology-based interventions are currently limited and contradictory (Sandford et al., 2006). For example, a large-scale study by the US Department of Education that compared three technology-based maths interventions in sixth Grade pupils across the US reported no significant improvement in test scores over a school year compared to pupils that had received normal instructional practice. Furthermore, differences in test scores were not related to any of the school and classroom characteristics measured (Dynarski et al., 2007). Yet young children appear to have a natural affinity toward understanding number concepts. Studies have shown that even babies can discriminate sets of objects that vary in number (Lipton and Spelke, 2003). Also, when children begin primary school they often come with an enthusiasm for learning, which if captured and supported, could propel them toward reaching their full potential. Thus, well-designed technologies that optimize learning could serve to support development of mathematical ability in the early primary years and provide a solid foundation on which to build further, more complex, abilities. Promoting understanding of mathematics in the early primary years is critical, as longitudinal research has shown that early mathematical understanding is highly influential on later mathematics and reading attainment at school (Duncan et al., 2007), even after controlling for other basic skills that are known to impact on scholastic attainment (Siegler et al., 2012).

According to the UNESCO-IBE (2010)^1^ standards in mathematics across Malawi are very poor. In tests conducted in 2004, 98% of pupils in Malawi did not possess mathematical skills beyond basic numeracy and Malawi was ranked the second lowest for mathematics of the 14 participating countries in the report. Similar findings were reported by USAID in tests conducted across 50 primary schools in Malawi in 2010^2^. The USAID report stated that children could “only answer the most elementary and procedural of items with any sense of confidence” (p. 42). Thus radical shifts in the teaching of mathematics are needed in Malawi to raise academic standards.

Recent advances in digital technology could provide an alternative or additional means of support to current classroom practices if technology-based interventions are shown to be effective. Mobile technologies, such as tablets, might be particularly suited to developing countries like Malawi, where class sizes are typically large (average 76 primary school children per class in Malawi in 2011 according to the UNESCO Institute for Statistics^3^) and the number of classrooms is usually small, leading to severe overcrowding. Classes are based on ability (Standard) rather than age and repetition rates are very high in Malawi, as many children fail to progress over a school year, so are required to take the year again. Typically, girls fare less well than boys in primary education in Malawi, with many dropping out of school by age 8 years. This results in a long-lasting gender disparity that affects economic growth in multiple ways. In addition, the quality of teaching in Malawi can vary greatly and many teachers may have received poor or little training. Coupled with few resources to assist with teaching, the learning environment is often extremely impoverished. Consequently, within the current primary school system in Malawi it is difficult to track developmental progression of individual children, identify those that are falling behind, and provide child-centered individual tuition at a consistent level of quality.

Digital technologies, such as tablets, afford many advantages to both the class teacher and their pupils, if the software is well-designed and the content is grounded in a solid well-constructed curriculum that is appropriate for the child’s developmental stage (Kucirkova, 2014). Even in crowded or outside classrooms, mobile technologies, such as tablets, can deliver one-to-one interactive instruction, with clear objectives, in a consistent manner to all children, thus equating teaching quality across pupils. Children can repeat material as often as they need, thus the pace of learning is tailored to individual needs. Individual progress can also be monitored objectively and easily, using assessments built into the software. With overcrowded classes and limited resources in Malawi, tablet-based interventions could help to radically improve academic standards across the primary years.

Yet despite the affordances of mobile technologies to support learning, even in the early years, there is a dearth of research providing an evidence base to justify their use in an educational context. A recent meta-analysis that examined 39 studies published between 1990 and 2012 reporting on the use of technological games in an educational context showed that technological game-based approaches were significantly more effective than typical instructional methods in improving learning (Wouters et al., 2013). Few studies have evaluated the use of technology-based educational games in supporting maths development in early education. Only three studies have been published to date that have examined the use of technology-based educational maths games with children in the first 3 years of primary school, which are reported in sufficient detail to allow objective comparison using effect sizes (Cohen, 1988). Across these studies, learning effects vary in extent from large (Cohen’s *d* > 0.8, Praet and Desoete, 2014) to medium (Cohen’s *d* > 0.5, Shin et al., 2012) or small-medium (Cohen’s *d* > 0.4, Räsänen et al., 2009). Each of these studies was conducted with European or North American children. Whilst effect sizes vary across studies, in general, these studies demonstrate the positive impact of technology-based educational games in supporting mathematical development in young children compared to standard practice. It remains to be determined if technology-based interventions can also be effective at supporting acquisition of early mathematical skills in developing countries, given the difficulties entrenched in the educational systems of countries such as Malawi, as outlined above.

This study reports the first randomized control trial (RCT) to evaluate the effectiveness of a tablet-based intervention to support mathematical ability in primary school children in Malawi. onebillion©, a not-for-profit publishing company, developed the tablet-based maths intervention that was evaluated. The intervention is currently being piloted in several primary schools in Malawi. Based on the National Primary Curriculum for mathematics delivered throughout primary schools in Malawi it is given in Chichewa – the official language in Malawi – via individual electronic tablets (Apple© iPad minis) and incorporates all of the features of well-designed software outlined above. The main aims of this study were to: (i) evaluate the effectiveness of the maths tablet intervention over normal classroom practice in supporting the development of mathematical ability in primary school children; (ii) establish the most appropriate Standard in which to implement the maths tablet intervention; and (iii) determine if girls respond differently to the maths tablet intervention than boys.

## Materials and Methods

### Design

A RTC was conducted in which children from Standards 1–3 at a medium-sized primary school (Biwi Primary School) situated in an urban area of Lilongwe, the capital city of Malawi, were randomized to one of three groups: maths tablet intervention (treatment group); non-maths tablet control (placebo group); and normal practice (control group). Children were tested on mathematical ability and basic skills associated with scholastic progression immediately before (pre-test) and after (post-test) the 8-weeks intervention period.

The Ministry of Education in Malawi gave consent for the study to take place and selected Biwi Primary School as the test school in Lilongwe. Prior to study commencement, consent was also gained from the parent association at Biwi Primary School and the Community Chief of the region where Biwi Primary School is located.

### Participants

**Table 1** summarizes the composition of the study sample at each stage of the RCT. In total, 350 children were enrolled to the study. These were all children from Standards 1–3 attending Biwi Primary School on the first 2 days of the 2013–2014 school year. Due to hardware constraints on the tablet interventions group size, 32 children were randomly excluded from the study. The remaining 318 children were randomized to one of three groups. Allocation to group was randomized at the class/gender level (i.e., within classroom randomization across gender). This was to ensure, as far as possible, an equal representation of boys and girls in each of the groups and to control for teacher effects, which is important given that class teachers implemented the interventions. Of the 318 children that were randomized to group, 304 children were pre-tested, using a tablet-based assessment app developed specifically for this study. Children that were randomized to group but not pre-tested (14 children in total) were either absent (six children) or had transferred school (eight children) when the pre-testing took place.

**Table 1:** Composition of the study sample at each stage of the RCT.

Study phase		Number of children	
**Enrolment**
Eligible (all children in S1–S3 attending school on first and second day of school year)		350 (S1 = 73; S2 = 125; S3 = 152)
Randomized (to one of three groups)		318 (S1 = 49; S2 = 125; S3 = 144)
Excluded (due to group size study design constraints)		32 (S1 = 24; S2 = 0; S3 = 8)
**Allocation**
**Group**	**Maths tablet**	**Non-maths tablet**	**Normal practice**

Randomized to group	115 S1 = 25; S2 = 42; S3 = 48	90 S1 = 0; S2 = 42; S3 = 48	113 S1 = 24; S2 = 41; S3 = 48
Pretested	112 S1 = 24; S2 = 41; S3 = 47	83 S1 = NA; S2 = 37; S3 = 46	109 S1 = 23; S2 = 40; S3 = 46
Not pretested (i.e., absent at pre-test)	1 S1 = 0; S2 = 1; S3 = 0	2 S1 = NA; S2 = 2; S3 = 0	3 S1 = 0; S2 = 1; S3 = 2
Received intervention	113 S1 = 24; S2 = 42; S3 = 47	85 S1 = NA; S2 = 39; S3 = 46	112 S1 = 23; S2 = 41; S3 = 48
Did not receive intervention (i.e., transferred before pre-test)	2 S1 = 1; S2 = 0; S3 = 1	5 S1 = NA; S2 = 3; S3 = 2	1 S1 = 1; S2 = 0; S3 = 0
**Follow up**
Post-tested	105 S1 = 22; S2 = 39; S3 = 44	82 S1 = NA; S2 = 38; S3 = 44	103 S1 = 20; S2 = 38; S3 = 45
Lost to follow up (i.e., absent or transferred by post-test)	8 S1 = 2; S2 = 3; S3 = 3	4 S1 = NA; S2 = 2; S3 = 2	9 S1 = 3; S2 = 3; S3 = 3
**Analyzed**
Excluded from analysis (i.e., absent at pre-test or post-test or transferred)	11 (9.6%) S1 = 3; S2 = 4; S3 = 4	11 (12.2%) S1 = NA; S2 = 7; S3 = 4	13 (11.5) S1 = 4; S2 = 4; S3 = 5
**Final sample** (i.e., present at pre-test and post-test)	**104** **S1 = 22; S2 = 38; S3 = 44**	**79** **S1 = NA; S2 = 35; S3 = 44**	**100** **S1 = 20; S2 = 37; S3 = 43**

In total, 113 children from Standards 1–3 received the tablet-based maths intervention and 112 children from Standards 1–3 received normal classroom practice during the 8-weeks intervention period. Additionally, 85 children from Standards 2–3 received a tablet-based control intervention that did not involve the maths software. This group was critical as it served as a placebo group to enable the generic effects of using tablets over the specific effects of the onebillion© maths software to be differentiated. In addition, this placebo intervention controlled for additional factors that might influence performance compared to normal classroom practice. For example, the tablet interventions were delivered in a small classroom to groups of 25 children (see Location of Interventions) whereas within the normal classroom practice maths instruction was delivered in a large classroom to groups of 70–80 children. Children in the tablet interventions (treatment and placebo) were thus singled out from the rest of the class whilst receiving the intervention, so received additional attention compared to their normal practice peers. Children taking part in the tablet interventions (treatment and placebo) also had more practice at using the device compared to children in the normal practice group. As two of the three assessments of mathematical ability given at post-test were delivered via the tablets, this placebo group was thus crucial in distinguishing specific effects of the maths software from these other extraneous variables associated with the tablet interventions that could influence performance. As such, this placebo group served to control against novelty and/or Hawthorne effects. Due to the limited numbers of children enrolled in Standard 1 at the start of the school year, Standard 1 children were not allocated to this non-maths tablet control intervention.

For all of the analyses below, a final sample was determined which included all children from Standards 1–3 (*n* = 283) that were present at both pre-test and post-test. **Table 2** gives the descriptive statistics of this final sample.

**Table 2:** Descriptive statistics of the final sample.

Standard	Group			
	Maths tablet	Non-maths tablet	Normal practice
**S1 (*n* = 42)**	*n* = 22	NA	*n* = 20
Age (months)	84 (10.8) 73–118		81 (4.5) 74–93
Gender (*F:M*)	10:12		5:15
**S2 (*n* = 110)**	*n* = 38	*n* = 35	*n* = 37
Age (months)	91 (8.7) 74–109	91 (9.5) 79–119	92 (10.7) 75–125
Gender (*F:M*)	18:20	19:16	17:20
**S3 (*n* = 131)**	*n* = 44	*n* = 44	*n* = 43
Age (months)	106 (15.9) 87–161	109 (13.4) 87–147	105 (14.2) 79–139
Gender (*F:M*)	22:22	24:20	24:19


*Age [mean (SD) and minimum–maximum] and gender (number of females F and males M) are reported for each of the standards assessed.*

### Groups

Children from Standards 1–3 were randomly allocated to one of the following groups.

#### Maths Tablet Intervention

This intervention consisted of four different apps developed by onebillion©: Masamu (Chichewa for Maths) 1, Masamu 2, Count to 10, and Count to 20. The apps are based on the National Primary Curriculum that is delivered in Malawi and teach core mathematical concepts (MCs) in a structured manner through several colorful and engaging sets of activities delivered in the local language, Chichewa. Children worked through the apps at their own pace and could practice particular activities as often as they desired. To progress to the next set of activities, children needed to ‘pass’ a quiz built into the software that assessed knowledge of the set of activities the child had been working on. Teachers monitored progress of individual children through achievement charts, in which a star was awarded to each child as they passed a particular quiz. Passing a quiz required 100% accuracy, thus thoroughly assessing children’s progression through the apps.

#### Non-Maths Tablet Control Intervention

This placebo intervention consisted of four different apps that are freely available to download from the Internet: Music Sparkles developed by Kids Game Club©, Drawing Pad developed by Darren Murtha Design©, and Toca Tailor and Toca Hair Salon developed by Toca Boca AB©. These apps were chosen because they are educational (supporting musical ability and design skills), receive good customer ratings, are non-verbal, and do not involve concepts taught in the maths tablet intervention. Additionally, these apps require children to interact with the tablet in terms of manual and attentional processes in a similar manner to the onebillion© maths apps, thus equating, as far as possible, practice of manual and attentional skills across the two tablet groups. Throughout the intervention period, children were free to choose whichever app they wanted, as often as they wanted.

#### Normal Practice

This consisted of normal instructional practice delivered in primary schools across Malawi. Children followed the Malawi National Primary Curriculum, delivered in Chichewa by their class teacher. Basic numeracy (including mathematics) is taught from Standard 1. Delivery was face-to-face with the class teacher giving maths instruction through the aide of a chalkboard, to a group of children sat on the floor. Most of the children had small notebooks and pencils in which to practice particular maths questions, written on the board or dictated orally (for classes taken outside) by the class teacher. For example, the teacher might write the following sum on the chalkboard, 7+2, and wait whilst the children completed this, in their notebooks. The children would then bring their notebooks to the teacher for their answer to be marked.

#### Comparison of Instruction Content and Delivery Across Groups

As the maths tablet intervention is also based on the Malawi National Primary Curriculum, children in each of the groups were exposed to the same basic curriculum. However, the different modes of implementation (tablet versus face-to-face) resulted in instructional differences, as both the content and delivery of teaching mathematics varied across the groups. For example, the tablet technology afforded individual child-centered learning so each child in the maths tablet intervention could work through the program at their own pace. Accordingly, the amount of time spent on particular topics varied across individuals within the maths tablet group, as did the rate of progression across the program. Children also received feedback through their interactions with the maths software. Positive feedback was received immediately upon successful completion of an item, after which the software introduced the next item to the child. In contrast, with normal classroom practice, the class teacher determined pace of delivery and amount of time spent on particular topics, and teaching was delivered to a large group of children (∼70–80 children). Class teachers gave feedback to individual children when they presented their response to the teacher after completing a particular question posed. However, compared to the maths software intervention, feedback per item to individual children was delayed in the standard instructional practice, as class teachers simply could not reach individual children as they completed a particular task. Although both of the maths interventions followed the same curriculum, the content was specific to each intervention. In the maths tablet intervention the content was consistent for all children and had been written by a well-reputed maths author. In standard classroom practice, the class teacher determined the content, as there was not a standard maths text that was followed.

#### Location of Interventions

Both of the tablet-based interventions (treatment and placebo) were administered in a purpose-built ‘Learning Centre’; a small classroom within the grounds of Biwi Primary School but detached from the main school buildings. The Learning Centre housed up to 25 children at a time, sat on bamboo mats, individually using tablets connected to personal headsets. A class teacher was present in the Learning Centre during the tablet-based interventions primarily to assist the children with using the technology. The Malawi branch of VSO provided additional technical support to class teachers throughout the intervention period. Class teachers responsible for overseeing the tablet-based interventions were given a training session prior to the intervention starting, in which they were shown how to use the tablets including turning the tablets on and off, connecting the headsets, storing the headsets, charging and securely storing the tablets, and navigating through the software. They were also shown how to record attendance and progress with the maths software for individual children throughout the intervention period using attendance/achievement charts specifically designed for this study that were pinned to a board in the Learning Centre. Children allocated to the normal practice intervention received the National Primary Curriculum in their usual classroom setting.

### Assessments

Tablet technology was used in the assessment of individual children on tasks of mathematical ability and basic skills as it enabled performance to be measured objectively, with large groups of children, within a short period of time. Accordingly, a new assessment app was developed specifically for this study with instructions given in Chichewa. The app consisted of two measures of mathematical ability and six measures of basic skills known to be associated with scholastic progression, namely manual processing speed, manual coordination, visual attention, short term memory, working memory, and spatial intelligence (Wei et al., 2011). All of these measures were designed by the author and were programmed by onebillion©. An introduction task taught children how to perform the critical operations required to complete the tasks in the assessment app, including how to select objects on the screen varying in size and how to select and move objects around the screen. Specific details of the tasks assessing basic skills, including measures of reliability and validity, are the focus of separate paper (see Pitchford and Outhwaite, in preparation).

#### Mathematical Ability

Two measures of mathematical ability were developed for the tablet-based assessment app Maths curriculum knowledge (CK) consisted of 50 quiz items taken from the Masamu 1 and Masamu 2 apps and thus assessed CK that is specific to the maths intervention. MCs consisted of 48 questions assessing a conceptual understanding of mathematics, similar to that used in the Brombacher (2011) study and the Numerical Operations subtest of the WIAT-II (Wechsler, 2005). Concepts assessed in this task included symbolic understanding, numbers in relation to each other, number line understanding, counting, number sense (quantity estimation), simple and complex addition, simple and complex subtraction, and multiplication and division. For both of these measures items were presented in a set order that increased with difficulty over successful trials. Accordingly, a discontinue rule was applied so that the task terminated after a specified number of consecutive fails. This prevented children becoming disengaged by having to answer questions that were beyond their ability.

To assess generalization of CK learned through the tablet-based intervention to a more conventional paper and pencil context a third measure of mathematical ability was developed. Maths curriculum knowledge generalization (CKG) was an additional measure given at post-test only, that included 50 new items based on those used in the maths tablet intervention, that the children had not seen before. Paper tests were prepared for each child, with their photograph and study number on the front page to ease identification of individual children. These were administered in groups of 25 children in the Learning Centre, with an assistant pointing to each question as a class teacher read out the question in Chichewa.

### Procedure

The study commenced in September 2013 on the first day of the school year and continued for a total of 10 weeks. Enrolment and pretesting was carried out over the first week by the author and three assistants (a VSO volunteer and a programmer and a translator from onebillion©). The intervention took place over the following 8 weeks and was implemented by class teachers at Biwi Primary School, with technical support by VSO. Post-test assessments were then conducted in November 2013, in week 10, by the author and two assistants (the VSO volunteer and the programmer from onebillion©).

#### Enrolment

Over the first 2 days of the school year all children that were eligible for the study were enrolled, randomly allocated to one of the three groups, and assigned a study number. Enrolment included photographing each child so as to ease identification of individual children assigned to the different groups. Gender, Standard, and Classroom were also recorded for each child and date of birth was collected through school records. To ensure children were enrolled just once, a temporary mark was placed on the child’s hand to indicate they had been registered. A computer program, written by onebillion©, was used to automatically assign children randomly to one of the three groups, prior to children being given the pre-test assessments.

#### Pre-Test and Post-Test Assessments

Groups of up to 50 children maximum were pre-tested or post-tested at the same time, in a regular classroom in the main school building. Prior to assessment, the tablets were set up with each child’s photograph, study number, and intervention group. This aided conducting the pre-test and post-test assessments as the tablets could be handed out reliably to individual children and their data was stored automatically to their allocated group. Children were tested per group (e.g., Standard 1 maths tablet intervention) where possible. Children were collected from their classroom by one of the assistants and escorted to the classroom where the assessments were carried out. When entering the assessment classroom the researchers (author) or assistant handed each child the tablet set up specifically for them and a headset. Children sat on the floor to carry out the assessments. The researcher stood at the front of the group and demonstrated each task in the assessment app via a large tablet with instructions being orally translated into Chichewa by a class teacher. Children first completed a brief demonstration of how to touch and move objects on the tablet screen, so as to familiarize them with using the technology. They then carried out each task in the assessment app, in a specified order. To start each task children were required to swipe a large white dot on an introduction screen after which the first practice trial of that task was administered. Children worked through each task, in sequence, until they had completed all of the assessments. A large star appearing on the screen and exploding into smaller stars marked the end of the assessment app. Children took around 40–60 min to complete the assessment app, depending on their level of ability. Upon completion, children handed their tablet and headset to the researcher or assistants and returned to their class.

#### Interventions

The intervention period lasted 8 weeks (40 school days). Each of the tablet-based interventions (treatment and placebo) was administered for 20 days. Children from Standards 2–3 received the tablet-based intervention to which they had been allocated for 1 h on alternate school days (20 h maximum across the 8-weeks intervention period). Children from Standard 1 received the maths tablet intervention for 30 min on alternate school days (10 h maximum across the 8-weeks intervention period). Class teachers responsible for overseeing the tablet-based interventions drew up a timetable, in which one set of teachers was allocated to the maths tablet intervention and a second set of teachers was allocated to the non-maths tablet control intervention. As far as timetabling permitted, children from Standards 1–3 that were allocated to the maths tablet intervention group received the maths tablet intervention whilst children in the other two groups (non-maths tablet intervention and normal practice) received the standard maths instruction that is normal practice in Malawi. This was in an attempt to equate total time spent on maths education across the three groups.

The tablets used in this study were iPad Minis as these are a suitable size for young children to hold and interact with whilst seated on the floor. They also have the battery capacity required for implementing this intervention on a daily basis. onebillion© placed 50 iPads into Biwi Primary School specifically for this study. This enabled 25 iPads to be used for the intervention on alternate days whilst the other 25 iPads were being charged. To ensure children received the correct intervention, 25 iPads with red covers had only the maths intervention software installed on them, and the other 25 iPads with blue covers had only the non-maths software installed on them. Furthermore, for the maths intervention group, iPads were personalized to individual children using their photographs and study number allocated at enrolment. This ensured that each child used the same iPad throughout the intervention period and allowed electronic data to be recovered on individual usage. Class teachers overseeing the tablet-based interventions were responsible for charging the iPads when not in use. At the end of the study, the maths tablet intervention was made accessible to all children from Standards 1–3 at Biwi Primary School, so as to enable each child to potentially benefit from this new educational technology resource.

### Data Analysis

For all of the analyses conducted the final sample of 283 children, reported in **Table 2**, was used. Mean performance was determined for individual children for each of the tasks given at pre-test and post-test. A combined measure of mathematical ability (mean of CK and MC) was also determined for each child by averaging scores across these two measures (maximum score = 49). To address the main aims of this study the following analyses were conducted.

(i) To evaluate the effectiveness of the maths tablet intervention over normal classroom practice in supporting the development of mathematical ability, mean performance for each of the groups was compared across time, at both pre-test and post-test, using mixed (where Time was the within-groups variable and Group was the between-groups variable). Analyses of variance (ANOVAs), for Standards 1–3, on each of the measures of mathematical ability. Separate 2 (Time: pre-test, post-test) × 3 (Group: maths tablet, non-maths tablet, normal practice) mixed ANOVAs were conducted for Standard 2 and Standard 3 and a 2 (Time: pre-test and post-test) × 2 (Group: maths tablet, normal practice) mixed ANOVA was conducted for Standard 1. In addition, for Standard 2 and Standard 3, separate one-way between-groups ANOVAs (Group: maths tablet, non-maths tablet, normal practice) were conducted to examine generalization of maths CK to a different paper and pencil format.
(ii) To establish the most appropriate Standard in which to implement the maths tablet intervention, between-group effect sizes (using Cohen’s *d*) were computed for each Standard at post-test for the maths tablet intervention group compared to the normal practice control group. Effect sizes provide a standardized measure on which to compare the magnitude of the intervention effect thus enabling direct comparison across Standards. According to Cohen (1988) an effect size of 0.2 is considered small, 0.5 is considered medium, and 0.8 or above is considered large.
(iii) To determine if girls respond differently to the maths tablet intervention than boys, mean performance for the combined measure of mathematical ability of girls and boys receiving the maths tablet intervention was compared to that of girls and boys receiving normal practice at pre-test and post-test, using a 2 (within-groups variable Time: pre-test, post-test) × 2 (between-groups variable Group: maths tablet; normal practice) × 2 (between-groups variable Gender: girls, boys) mixed ANOVA.

## Results

Results from the analyses reported above are summarized in **Tables 3–5** below.

**Table 3:** Group performance [mean (SD), minimum–maximum] at pre-test and post-test and percentage performance gain for the tests of mathematical concepts (MCs; maximum score 48), Maths curriculum knowledge (CK; maximum score 50), and Maths curriculum knowledge generalization (CKG; maximum score 55).

Standard Assessment Time of test		Group		
		Maths tablet	Non-maths tablet	Normal practice
**S1 (*n* = 44)**		*n* = 22	NA	*n* = 20
**MC**	Pre	2.0 (1.7) 0–5		3.4 (4.3) 0–13
	Post	5.1 (4.6) 0–17		4.5 (4.3) 0–13
	***% Gain***	***6.5***		***2.3***
**CK**	Pre	2.4 (2.7) 0–12		2.7 (1.7) 0–6
	Post	7.7 (6.3) 0–24		5.9 (5.3) 0–20
	***% Gain***	***10.6***		***6.4***
**S2 (*n* = 110)**		*n* = 38	*n* = 35	*n* = 37
**MC**	Pre	8.6 (6.5) 0–20	10.1 (5.7) 0–21	8.4 (6.3) 0–19
	Post	14.6 (6.6) 0–24	12.5 (7.3) 0–23	10.5 (6.5) 0–19
	***% Gain***	***12.5***	***5.0***	***4.4***
**CK**	Pre	6.4 (6.3) 0–24	8.3 (8.1) 0–28	5.5 (5.3) 0–21
	Post	20.7 (10.3) 0–37	15.1 (8.5) 2–31	10.8 (7.4) 0–26
	***% Gain***	***28.6***	***13.6***	***10.6***
**CKG**	Post	23.8 (9.1) 7–41.5	17.4 (10.3) 0–36	20.7 (8.4) 4–39
**S3 (*n* = 131)**		*n* = 44	*n* = 44	*n* = 43
**MC**	Pre	14.9 (5.7) 0–23	15.4 (5.6) 0–22	14.9 (5.2) 0–22
	Post	19.5 (5.2) 5–27	19.3 (5.1) 4–26	18.6 (6.0) 0–26
	***% Gain***	***9.6***	***8.1***	***7.7***
**CK**	Pre	13.5 (9.3) 0–34	14.8 (10.1) 1–37	11.0 (10.3) 0–33
	Post	35.2 (7.0) 19–46	24.9 (8.0) 3–36	23.4 (6.9) 7–36
	***% Gain***	***43.4***	***20.2***	***24.8***
**CKG**	Post	33.5 (7.3) 19–47.5	20.0 (6.7) 7–36	27.2 (8.1) 10–43

**Table 4:** Between-group effect sizes (Cohen’s *d*) across Standards 1–3 for differences in mean performance of the maths tablet intervention group compared to the normal practice control group at post-test for the MCs, Maths CK, and Maths CKG test.

Standard	Measure		
	MC	CK	CKG
1	0.135	0.310	NA
2	0.626	1.119	0.354
3	0.161	1.698	0.818

**Table 5:** Performance [mean (SD) minimum–maximum] of girls and boys across Standards 1–3, at pre-test and post-test, on the combined measure of mathematical ability (maximum score 49).

Standard Time of test Gender		Group		
		Maths tablet	Normal practice
**S1 (*n* = 44)**		*n* = 22 (10:12)	*n* = 20 (5:15)
Pre-test	Girls	2.3 (1.4) 0.0–4.0	3.6 (3.0) 0.5–8.5
	Boys	2.1 (1.9) 0.5–6.0	2.9 (2.7) 0.0–9.5
	***Difference***	***0.2***	***0.7***
Post-test	Girls	7.4 (5.4) 0.5–15.5	5.5 (4.7) 0.0–10.0
	Boys	5.6 (3.8) 2.5–14.0	5.0 (3.9) 0.0–13.0
	***Difference***	***1.8***	***0.5***
**S2 (*n* = 110)**		*n* = 38 (18:20)	*n* = 37 (17:20)
Pre-test	Girls	6.3 (5.2) 0.0–20.5	6.0 (4.7) 0.0–14.5
	Boys	8.5 (6.3) 0.5–21.5	7.7 (5.4) 1.0–19.0
	***Difference***	***2.2***	***1.7***
Post-test	Girls	15.6 (8.5) 0.0–30.0	9.0 (6.1) 0.0–20.0
	Boys	19.5 (7.1) 5.0–30.0	12.1 (6.4) 0.5–22.5
	***Difference***	***3.9***	***3.1***
**S3 (*n* = 131)**		*n* = 44 (22:22)	*n* = 43 (24:19)
Pre-test	Girls	14.5 (6.9) 3.0–26.5	11.7 (4.4) 1.0–20.0
	Boys	13.9 (6.7) 0.0–27.5	14.6 (6.5) 1.5–25.5
	***Difference***	***0.6***	***2.9***
Post-test	Girls	28.3 (5.0) 16.5–35.0	20.4 (5.9) 4.0–26.5
	Boys	26.4 (5.9) 13.0–33.5	21.8 (4.4) 13.0–29.5
	***Difference***	***1.9***	***1.4***

*Total number of children given per Standard, followed by distribution of girls to boys.*

### Effectiveness of the Maths Tablet Intervention

**Table 3** reports the mean performance at pre-test and post-test for each of the groups across Standards 1–3 on the two measures of mathematical ability (MC and CK) included in the tablet-based assessment app and the paper and pencil generalization test (CKG) given at post-test.

#### Standard 1

For the test of MCs the 2 (Time: pre-test, post-test) × 2 (Group: maths tablet, normal practice) mixed ANOVA revealed a significant main effect of Time, *F*(1,40) = 7.2, *p* = 0.011, but not Group, *F*(1,40) = 0.189, *p* = 0.666, and no significant interaction between Time and Group, *F*(1,40) = 1.71, *p* = 0.198. Planned comparisons conducted within each of the groups across time showed that whilst the maths tablet group made significant gains over time, *t*(21) = 3.11, *p* = 0.005, the normal practice control group did not, *t*(19) = 0.89, *p* = 0.386.

Similar results were found with the test of CK. The 2 (Time: pre-test, post-test) × 2 (Group: maths tablet, normal practice) mixed ANOVA revealed a significant main effect of Time, *F*(1,40) = 24.15, *p* < 0.001, but not Group, *F*(1,40) = 0.524, *p* = 0.473, and no significant interaction between Time and Group, *F*(1,40) = 1.63, *p* = 0.21. For this measure of maths ability, planned comparisons showed that both groups made significant gains over time: maths tablet, *t*(21) = 4.17, *p* < 0.001; normal practice, *t*(19) = 2.77, *p* = 0.012.

#### Standard 2

For the test of MCs results from the 2 (Time: pre-test, post-test) × 3 (Group: maths tablet, non-maths tablet, normal practice) mixed ANOVA showed a significant main effect of Time, *F*(1,107) = 32.13, *p* < 0.001, no significant main effect of Group, *F*(2,107) = 1.54, *p* = 0.219, but a significant interaction between Time and Group, *F*(2,107) = 4.13, *p* = 0.019. Analysis of simple main effects at pre-test showed no significant effect of Group, *F*(2,107) = 0.82, *p* = 0.443, indicating that at pre-test the different groups were matched in MCs ability. At post-test, however, a significant main effect of Group was found, *F*(2,170) = 3.32, *p* = 0.039. *Post hoc* analyses with Bonferroni, corrected for multiple comparisons, revealed the maths tablet group significantly outperformed the normal practice control group, *p* = 0.033. No other comparisons were significant (maths tablet and non-maths tablet placebo: *p* = 0.585; non-maths tablet placebo and normal practice: *p* = 0.657). Over time, significant performance gains were found for the maths tablet intervention group, *t*(37) = 5.47, *p* < 0.001, and the normal practice control group, *t*(36) = 2.69, *p* = 0.011, but not for the non-maths tablet placebo group, *t*(34) = 1.86, *p* = 0.071.

With the test of maths CK, ANOVA results revealed a significant main effect of Time, *F*(1,107) = 147.24, *p* < 0.001, and a significant main effect of Group, *F*(2,107) = 6.12, *p* = 0.003. The Time × Group interaction was also significant, *F*(2,107) = 15.18, *p* < 0.001. Analysis of simple main effects at pre-test revealed no significant effect of Group, *F*(2,107) = 1.70, *p* = 0.188, indicating the groups were matched in CK prior to intervention. A significant main effect of Group was, however, found at post-test, *F*(2,107) = 12.03, *p* < 0.001. *Post hoc* tests with Bonferroni revealed the maths tablet group significantly outperformed both the normal practice control group, *p* < 0.001, and the non-maths tablet placebo group, *p* = 0.024. In contrast, the difference in performance at post-test between the two control groups was not significant, *p* = 0.115. Across the intervention period, all groups made significant gains in maths CK [maths tablet: *t*(37) = 9.78, *p* < 0.001; non-maths tablet placebo: *t*(34) = 5.57, *p* < 0.001; normal practice: *t*(36) = 5.16, *p* < 0.001].

In addition, one-way between-groups ANOVA (Group: maths tablet, non-maths tablet, normal practice), conducted on the CKG test administered at post-test, revealed a significant main effect, *F*(2,101) = 4.12, *p* = 0.019. *Post hoc* analyses with Bonferroni showed the maths tablet intervention group outperformed the non-maths tablet placebo group, *p* = 0.015, but the other pairwise comparisons did not reach significance (non-maths tablet and normal practice: *p* = 0.474; maths tablet and normal practice, *p* = 0.497).

#### Standard 3

For the test of MCs the 2 (Time: pre-test, post-test) × 3 (Group: maths tablet, non-maths tablet, normal practice) mixed ANOVA revealed a significant main effect of Time, *F*(1,117) = 56.91, *p* < 0.001. Collapsed over Group, post-test performance was higher than pre-test performance on this measure. However, neither the main effect of Group, *F*(2,117) = 0.19, *p* = 0.831, nor the Time × Group interaction, *F*(2,117) = 0.24, *p* = 0.785, was significant.

With the test of CK, ANOVA results showed significant main effects of Time, *F*(1,128) = 351.92, *p* < 0.001, and Group, *F*(2,128) = 10.29, *p* < 0.001, and a significant interaction between Time and Group, *F*(2,128) = 20.45, *p* < 0.001. Analysis of simple main effects at pre-test revealed no significant effect of Group, *F*(2,128) = 1.68, *p* = 0.191, illustrating the groups were matched prior to intervention. However, at post-test a significant effect of Group was found, *F*(2,128) = 34.25, *p* < 0.001. *Post hoc* tests with Bonferroni showed the maths tablet group outperformed both the normal practice control group, *p* < 0.001, and the non-maths tablet placebo group, *p* < 0.001. In contrast, no significant different was found between the two control groups (non-maths tablet and normal practice, *p* = 0.996). Over time, all groups made significant improvements on the maths CK test [maths tablet: *t*(42) = 17.297, *p* < 0.001; non-maths tablet: *t*(37) = 6.849, *p* < 0.001; normal practice: *t*(38) = 8.670, *p* < 0.001].

These effects of the maths tablet intervention on CK generalized to paper and pencil format at post-test. One-way between-groups ANOVA on the CKG test revealed a significant main effect of Group, *F*(2,129) = 0.36.32, *p* < 0.001. *Post hoc* analyses with Bonferroni showed that maths tablet intervention group significantly outperformed both the normal practice control group, *p* < 0.001, and non-maths tablet placebo group, *p* < 0.001. The non-maths tablet placebo group also performed significantly poorer than the normal practice control group on this generalization test, *p* < 0.001.

#### Summary and Discussion

Results from the ANOVAs conducted for each Standard revealed the maths tablet intervention group showed: (1) Significantly greater gains in performance over time compared to the normal practice control group on the MCs test for Standard 2 children. (2) Significantly greater gains in performance over time compared to both the non-maths tablet placebo group and the normal practice control group on the Maths CK test for Standard 2 and Standard 3 children. (3) Significantly higher performance on the paper and pencil generalization test (CKG) at post-test than the non-maths tablet placebo group for Standard 2 children, and both the non-maths tablet placebo group and normal practice control group for Standard 3 children.

Whilst clear benefits were found after using the maths tablet intervention for Standard 3 children, results for Standard 2 children are somewhat mixed. For Standard 2 children on the test of MCs, although mean performance at post-test was higher for the maths tablet group (14.6) than the non-maths tablet placebo group (12.5), this difference did not reach significance. Despite this, over the 8-weeks intervention period, the maths tablet group made more improvement (12.5%) than the non-maths tablet placebo control (5%) whose performance gains was similar to the normal practice control group (4.4%). This arises from the higher level of performance at pre-test on the MCs test for the non-maths tablet placebo group (10.1) compared to both the maths tablet group (8.6) and the normal practice control group (8.4). Accordingly, within-groups effect sizes computed across the intervention period were large for the maths tablet group (Cohen’s *d* = 0.916) but small for both the non-maths tablet placebo group (Cohen’s *d* = 0.381) and the normal practice control group (Cohen’s *d* = 0.328). Likewise, for Standard 2 children on the Maths CKG test, there was no significant difference between the maths tablet group (23.8) and the normal practice control group (20.7), again probably due to limited power. Limited statistical power could also be the reason why no significant group differences were found for Standard 1 children at post-test, despite the maths tablet group making greater gains over time (6.5 and 10.6%) than the normal practice control group (2.3 and 6.4%) on the tests of MCs and Maths CK, respectively. This is reflected in larger effect sizes for the maths tablet group than the normal practice control group computed across the intervention period for both the MCs test (maths tablet group Cohen’s *d* = 0.894; normal practice control group Cohen’s *d* = 0.256) and Maths CK test (maths tablet group Cohen’s *d* = 1.094; normal practice control group Cohen’s *d* = 0.813).

Overall, these results indicate that intervention with the tablet-based maths software can add significant value to normal classroom practice in Malawi at supporting development of mathematical skills in primary school children. Dedicated time spent interacting with tablet technology in small groups away from the rest of the class is insufficient to support mathematical skills, as the gains made by the non-maths tablet placebo group did not differ significantly from the normal practice control group. In general, compared to normal practice, intervention with the tablet-based maths software seems to support a conceptual understanding of mathematics in Standard 2 and more specific curriculum-based knowledge in Standards 2 and 3, which generalizes to different formats by Standard 3, as shown by the paper and pencil test.

### Optimal Standard to Implement Maths Tablet Intervention

Effect size analyses conducted at post-test across the maths tablet intervention group and the normal practice control group are reported in **Table 4**. As can be seen, the largest effect of the maths tablet intervention was found with Standard 2 children for the MCs test compared to Standard 1 and Standard 3 children. Standard 2 children also showed a very large effect of the maths tablet intervention for the Maths CK test although the effect size was even larger for Standard 3 children for this measure. Additionally, Standard 3 children showed the largest effect size of the maths tablet intervention for the paper and pencil generalization test (CGK).

#### Summary and Discussion

These results suggest that implementing the maths tablet intervention in Standard 2 is optimal for supporting development of both conceptual and specific curriculum-based mathematical abilities. It is important to note, however, that Standard 1 children received 20 h of intervention with the maths software whereas children in Standards 2 and 3 received 40 h. Although the effect sizes in Standard 1 were small (MC) to small-to-medium (MQ), it is possible that larger effects of the maths tablet intervention may be obtained by increasing the time on task in Standard 1.

### Gender Differences

Using the combined measure of mathematical ability (maximum mean score 49), mean performance for girls and boys in each Standard, at pre-test and post-test, was determined. Results are reported in **Table 5**.

A 2 (Time: pre-test, post-test) × 2 (Group: maths tablet, normal practice) × 2 (Gender: girls, boys) mixed ANOVA was conducted to explore if girls responded differently to boys to the maths tablet intervention. Results showed no significant main effect of Gender, *F*(1,200) = 0.07, *p* = 0.799, and no significant interactions with Gender across Time, *F*(1,200) = 0.96, *p* = 0.329, and across Group, *F*(1,200) = 0.02, *p* = 0.799. The three-way interaction between Time, Group and Gender was also not significant, *F*(1,200) = 0.13, *p* = 0.720. Consistent with the analyses reported above, significant main effects of Time, *F*(1,200) = 348.81, *p* < 0.001, and Group, *F*(1,200) = 7.17, *p* = 0.008, were found, as was the interaction between Time and Group, *F*(1,200) = 34.52, *p* < 0.001.

#### Summary and Discussion

These results suggest that both girls and boys responded similarly to the maths tablet intervention and normal practice over time. As performance gains by children receiving the maths tablet intervention were significantly higher than those exposed to normal classroom practice, this analysis shows that the maths tablet intervention is just as effective for girls as it is for boys. Independent three-way mixed ANOVAs conducted on the separate measures of MCs and CK confirmed there were no significant gender effects in this sample.

## Discussion

This study reports the first RCT to be conducted to evaluate the effectiveness of a new maths tablet intervention currently being trialed in primary schools in Malawi. To differentiate the effectiveness of the maths software from the tablet hardware and small group setting of the Learning Centre built to administer the tablet intervention, this RCT comprised of three treatment arms: the maths tablet intervention delivered in the Learning Centre, a non-maths tablet placebo intervention also delivered in the Learning Centre, and a normal classroom practice control delivered in the typical classroom setting. Ecological validity was ensured through the tablet interventions being administered by class teachers, with technical support being provided by a local VSO volunteer. This represents a sustainable model for scale-up. Baseline measures of mathematical ability were taken prior to the intervention period. These consisted of assessments of conceptual understanding of maths and specific curriculum-based mathematical knowledge. The intervention period lasted 8 weeks, with children receiving the tablet-based interventions for the equivalent of 30 min a day. Children were reassessed on the measures of mathematical ability at the end of the 8-weeks intervention period so that learning gains could be determined.

Results clearly showed that the maths tablet intervention was significantly more effective at improving mathematical attainment than current instructional practice for primary school children in Malawi. First, Standard 2 children receiving the maths tablet intervention made significantly greater gains in performance over time (12.5%) compared to children receiving normal classroom practice (4.4%) on a measure of MCs. (Note: although the non-maths tablet placebo group only made a 5% increase over time on this test, their performance at post-test did not differ significantly from the maths tablet group) As this measure was comprised of items that were not used in any of the interventions, it illustrates genuine advances in mathematical understanding by Standard 2 children after interacting with the maths tablet intervention. Second, whilst all groups made significant improvements in CK over time, children receiving the tablet-based maths intervention showed greater performance gains than the control children, and this was significant for children in Standards 2 and 3. To some extent this might be expected, as children in the maths tablet intervention received some practice with the items included in the maths curriculum test, as these were drawn from the quizzes used in the maths tablet intervention. Thus, they would have been exposed to these items at least once whilst interacting with the software, if they had completed the maths intervention program in full. In addition, this test was administered via tablets, so familiarity with using the devices might have advantaged children receiving the maths tablet intervention over children receiving normal classroom practice. However, the non-maths tablet placebo group was incorporated into this RCT design specifically to guard against effects of enhanced familiarity with tablet use, as well as other key factors that might influence performance (such as small versus large group teaching). Results showed no advantage for the non-maths tablet intervention placebo group over the normal practice control group in maths CK, indicating that the tablet mode of delivery of this test cannot adequately account for the pattern of results found. Furthermore, the maths CK acquired by children in Standard 3 through interaction with the maths tablet intervention generalized to novel items, that were not practiced during the maths tablet intervention, and were delivered in a different, paper and pencil, format. This test of generalization was included to guard against practice effects with specific items in the maths tablet intervention, and thus shows that the higher levels of CK observed by Standard 3 children receiving the maths tablet intervention was unlikely to arise from practice effects with specific items used in the intervention. Despite this, the primary use of tablet technology to assess maths performance at post-test can be considered a shortcoming of the present study. Future studies should include additional tests of generalization to novel items and formats. Measures of application of new mathematical knowledge gained through the tablet intervention to real life contexts could be particularly useful to establish.

Importantly, the enhanced learning gains found by children interacting with the maths tablet intervention can be attributed to the maths software rather than the tablet hardware and small group setting of the Learning Centre, as children who received the critical non-maths tablet placebo intervention did not make significant gains in performance relative to the control children exposed to normal classroom teaching. The non-maths tablet intervention placebo group was incorporated into this RCT design to guard against other factors, such as size and location of class, that differed across the maths tablet intervention group and the normal practice control group, from influencing results. This placebo group also controlled for enhanced familiarity with using tablets, which might have affected performance on the tablet-based assessments at post-test. Importantly, a paper and pencil test assessing generalization of CK administered at post-test, showed significantly enhanced CK on novel, unseen items, by Standard 3 children who had received the maths tablet intervention compared to both normal practice and non-maths tablet controls. This shows that familiarity with tablets cannot account for the enhanced learning gains obtained through the tablet technology by Standard 3 children. Rather, the results illustrate that learning had been embedded within the children’s cognitive systems, allowing them to generalize this knowledge to a new, paper and pencil, context. Thus, the mechanism for supporting the substantial learning gains reported in this study focuses on the engaging, child-centered, curriculum-based, maths software, rather than the tablet technology and small group setting *per se*. This supports the assertion of Kucirkova (2014) that, with well-designed software that is grounded in an evidence-based curriculum that is appropriate for the child’s developmental stage, digital technologies can provide a useful classroom aide for supporting acquisition of basic skills.

It is important to note that time on task was equated, as far as possible, across group within each Standard. Thus, the enhanced learning gains found by Standards 2 and 3 children after interacting with the maths software compared to children receiving normal classroom practice are most likely to be attributable to the software itself. This might be due to the immediate feedback and active retrieval features that are built into the onebillion© maths software. As described previously, for each item in the software feedback is given to the child as they interact with the program. Within each item, feedback is given by way of a ‘negative’ sound when an error is made and a ‘positive’ sound when a correct action is performed. Some items require several actions to be performed for completion. Successful completion of an item is marked by the appearance of a big yellow tick and a ‘positive’ sound. This interactive feature of the software is clearly not possible in normal classroom practice where face-to-face delivery is to large groups of children. In addition, progression through the maths software is dependent upon successful completion of each item in set sequence of delivery that is graded in terms of difficulty. As a child must complete an item successfully before they can progress to the next item, this ensures graded learning that is firmly embedded within their cognitive system. Furthermore, a block of items that train a particular concept must be completed prior to progressing to another block of items training a different, more difficult, concept. At the end of a set of nine blocks of items, each block training a different concept, children complete a quiz that assesses their knowledge of the concepts trained over the previous nine blocks. This engenders ‘retrieval-based’ learning which is known to enhance not only encoding of new information but also the application of knowledge (e.g., Grimaldi and Karpicke, 2014). Thus, the interactive feedback and active retrieval features that are built into the onebillion© maths software are most likely to be the mechanisms that underpin generalization of maths knowledge shown by Standards 2 and 3 children to different performance measures (i.e., MCs and new CK) and modes of delivery (i.e., pencil and paper format).

In many developing countries, such as Malawi, delivery of standard instructional practice is dependent on various factors, such as weather (which determines if classes take place indoors or outside) and teacher availability (which depends on whether or not they have been paid, external family matters which can be frequent and significant, etc.). Consequently, daily timetables are highly flexible. This means that when conducting school-based studies in developing countries facing these conditions, it is not possible to quantify the precise time that each child has spent learning different subjects delivered through standard practice. Although attempts were made in this study to equate time-on-task across the different groups, it is not possible to establish how much time each child spent learning maths or any other subject through normal classroom practice. Only in the maths tablet intervention was time-on-task measured objectively as the tablet technology affords precise monitoring of time-on-task to be recorded. This can be seen as an additional benefit of using tablet technology in developing countries, such as Malawi, where is it presently very difficult to assess the amount of instruction a child has received in any given subject. Being able to monitor objectively time-on-task will enable studies to assess the relationship between duration of intervention and individual children’s progression, which will lead to greater efficiency in implementing educational interventions. Furthermore, this study has illustrated that tablet technology can be implemented readily within the standard primary school timetable to support development of basic skills. This gives teachers an opportunity to support individual children’s development, without the need to increase teachers’ time in class. Hence, tablet technology can be used effectively to increase overall school efficiency, thus justifying the costs associated with equipping schools with this technology.

Effect size analysis conducted across ability levels suggested that the maths tablet intervention was most effective at supporting conceptual understanding of mathematics when implemented in Standard 2 (Cohen’s *d* = 0.626) and specific curriculum-based mathematical knowledge when implemented in Standard 3 (Cohen’s *d* = 1.698). The large effect sizes found in this study for the children exposed to the maths tablet intervention exceed, or are comparable to, those reported by other studies in the literature (Räsänen et al., 2009; Shin et al., 2012; Praet and Desoete, 2014) conducted with European and North American primary school children. This demonstrates that technology-based interventions can be just as effective, if not more, at supporting early mathematical skills in developing countries, such as Malawi, that face serious challenges within their educational system, as they can in developed societies where systemic challenges are not as significant.

Finally, a gender analysis showed that the maths tablet intervention was just as effective at supporting the development of early mathematical skills in girls as it is for boys. This is an important finding for countries, such as Malawi, where girls typically fare less well than boys in primary education, and many drop out of school by age 8 years. As outlined in the Introduction, this gender disparity currently persists throughout society in Malawi and hinders economic growth. The results from this study provide robust evidence to support the continuation of girls’ education across primary and secondary school, and further into the higher education sector.

The use of tablet technology, coupled with well-designed, curriculum-based, engaging software, that allows the child to work at their own pace, could help to radically transform scholastic progression and attainment in developing countries, such as Malawi, that are currently facing significant educational challenges. With school days as short as 3 h for Standard 1 children in Malawi, fast learning gains are needed to optimize time spent at school. Tablet-based interventions that can be delivered in 30 min a day, that are shown to give rise to significantly enhanced meaningful learning, over a short period of time (e.g., 8 weeks, as in this study), could revolutionize early years education in developing countries, such as Malawi. However, when scaling up to a national level, careful deployment is required for these technologies to be optimized and deeply embedded within the educational system. For example, teachers need to be confident in using tablet technology within their everyday practice and they need to be aware of the potential that tablet technology can have in supporting learning. This will require investment in teacher training of both trainee teachers within teacher training institutions and existing teachers through continuing professional development courses. Technological support will be necessary to ensure maximal usage and uptake of tablet interventions. In developing countries, this could be implemented through capacity building exercises, such as training community-based volunteers, supported by organizations like VSO. Funds need to be available, not only for the initial investment in hardware but also for its maintenance and upgrading over time to ensure sustainability. Critically, software that is implemented to support the development of scholastic skills needs to be formally evaluated, through scientific studies such as this, in order to determine its effectiveness and potential to impact on educational attainment. Additional research could explore how to optimize tablet interventions, by manipulating the context in which they are used. For example, Johnson and Johnson (2014) consider how technology can enrich cooperative learning and enhance its effectiveness, which can promote not only educational attainment but also positive social interactions – a critical component of psychological well-being. This will require scientists working in partnership with local government, educators, businesses, charities and international non-government organizations, amongst others, to ensure that the investment required for nationwide scale up is optimized to support children’s learning. Only then will the true potential for tablet technology to revolutionize learning be realized.

## Conflict of Interest Statement

The Guest Associate Editor Robert Samuel Savage declares that, despite having collaborated on the Research Topic (Using technology to revolutionize learning: assessment, intervention, evaluation, and historical perspective) with author Nicola J. Pitchford, the review process was handled objectively. The author declares that the research was conducted in the absence of any commercial or financial relationships that could be construed as a potential conflict of interest.

## Footnotes

1 http://www.ibe.unesco.org

2 http://pdf.usaid.gov/pdf_docs/pnaec139.pdf

3 http://stats.uis.unesco.org
